# Modeled microgravity unravels the roles of mechanical forces in renal progenitor cell physiology

**DOI:** 10.1186/s13287-024-03633-3

**Published:** 2024-01-17

**Authors:** Maria Elena Melica, Francesca Cialdai, Gilda La Regina, Chiara Risaliti, Tommaso Dafichi, Anna Julie Peired, Paola Romagnani, Monica Monici, Laura Lasagni

**Affiliations:** 1 Department of Clinical and Experimental Biomedical Sciences “Mario Serio”, University of Florence, Viale Morgagni 50, 50134 Florence, Italy; 2 ASAcampus Joint Laboratory, ASA Res. Div., Department of Clinical and Experimental Biomedical Sciences “Mario Serio”, University of Florence, Viale G. Pieraccini 6, 50139 Florence, Italy; 3 Nephrology and Dialysis Unit, Meyer Children’s Hospital IRCCS, 50139 Florence, Italy

**Keywords:** Modeled microgravity, Renal progenitors, Podocyte, Cyclosporine A, Mechanobiology

## Abstract

**Background:**

The glomerulus is a highly complex system, composed of different interdependent cell types that are subjected to various mechanical stimuli. These stimuli regulate multiple cellular functions, and changes in these functions may contribute to tissue damage and disease progression. To date, our understanding of the mechanobiology of glomerular cells is limited, with most research focused on the adaptive response of podocytes. However, it is crucial to recognize the interdependence between podocytes and parietal epithelial cells, in particular with the progenitor subset, as it plays a critical role in various manifestations of glomerular diseases. This highlights the necessity to implement the analysis of the effects of mechanical stress on renal progenitor cells.

**Methods:**

Microgravity, modeled by Rotary Cell Culture System, has been employed as a system to investigate how renal progenitor cells respond to alterations in the mechanical cues within their microenvironment. Changes in cell phenotype, cytoskeleton organization, cell proliferation, cell adhesion and cell capacity for differentiation into podocytes were analyzed.

**Results:**

In modeled microgravity conditions, renal progenitor cells showed altered cytoskeleton and focal adhesion organization associated with a reduction in cell proliferation, cell adhesion and spreading capacity. Moreover, mechanical forces appeared to be essential for renal progenitor differentiation into podocytes. Indeed, when renal progenitors were exposed to a differentiative agent in modeled microgravity conditions, it impaired the acquisition of a complex podocyte-like F-actin cytoskeleton and the expression of specific podocyte markers, such as nephrin and nestin. Importantly, the stabilization of the cytoskeleton with a calcineurin inhibitor, cyclosporine A, rescued the differentiation of renal progenitor cells into podocytes in modeled microgravity conditions.

**Conclusions:**

Alterations in the organization of the renal progenitor cytoskeleton due to unloading conditions negatively affect the regenerative capacity of these cells. These findings strengthen the concept that changes in mechanical cues can initiate a pathophysiological process in the glomerulus, not only altering podocyte actin cytoskeleton, but also extending the detrimental effect to the renal progenitor population. This underscores the significance of the cytoskeleton as a druggable target for kidney diseases.

**Supplementary Information:**

The online version contains supplementary material available at 10.1186/s13287-024-03633-3.

## Background

The cytoskeleton constitutes a pivotal structure that confers shape and stability to the cell. It also functions as a complex sensor of mechano-biological inputs from the microenvironment and as a transductor of forces to the nucleus, influencing gene expression and various cellular functions, such as proliferation, and differentiation [1]. Among the 26 different cell types described in the kidney [2], podocytes possess a unique and intricate cytoskeleton. This cytoskeleton upholds their structural integrity and generates contractile forces to face the mechanical challenges posed by pulsatile blood flow and shear stress resulting from filtrate flows across foot processes [3]. In a nutshell, normal podocyte function depends on the maintenance of the actin cytoskeleton. Any structural changes in the actin fibers may potentially be responsible for pathologic changes in cell morphology [4, 5], such as foot process effacement. This, in turn, can result in podocyte detachment or death, directly impacting kidney function and causing proteinuria [5, 6]. Accordingly, direct stabilization of the actin cytoskeleton in kidney podocytes represents an attractive therapeutic approach [7]. Mechanical forces within the glomerulus also have an impact on other cell types including podocyte progenitors, which are a subset of parietal epithelial cells along the Bowman capsule able to supply new podocytes in response to glomerular injury [8–11]. While efficient podocyte regeneration by renal progenitor cells (RPCs) positively affects the health of the glomerular filtration barrier, this process is frequently inefficient, can drive focal scarring or lead to the generation of hyperplastic lesions known as crescent [4, 12]. These outcomes result from perturbation of the biochemical and biomechanical cues involved in the proliferative and differentiation programs. The demonstration that changes in matrix stiffness can alter cytoskeletal organization and affect the proliferation, migration and differentiation of RPCs toward the podocyte lineage was the first proof of the critical role exerted by mechanical cues in RPC behavior [13]. However, to date, the complexity of the mechanical phenomena, often referred to as “mechanomics”, involved in renal progenitor physiology remains poorly explored and understood.

A compelling body of evidence demonstrating how the cytoskeleton can capture and amplify even minor changes occurring in the field of force in numerous mammalian cells has emerged from biological research conducted in space and in microgravity (µ*g*) conditions reproduced on Earth (modeled µ*g*). Specific facilities, such as the monoaxial clinostats Rotating Wall Vessel (RWV) and Rotary Cell Culture System (RCCS), and the Random Positioning Machine (RPM) can be used to create µ*g* in research laboratories [14–17]. The majority of research in this field has primarily focused on specific cell types, such as osteoblasts/osteoclasts [18–20], myofibers [21], endothelial cells [22–24], stem cells [25, 26], fibroblasts [27–31] chondrocytes [32], lymphocytes [33] and other immune cells [34], with limited attention given to kidney cells [35, 36].

In this paper, we aim to exploit the unloading conditions generated by RCCS with the following purposes: (1) explore the effect of mechanical forces on RPC cytoskeleton organization; (2) evaluate the impact of cytoskeleton alteration on RPC capacity to differentiate into podocytes; (3) rescue the phenotype using actin-stabilizing drugs and (4) investigate the possibility to use this culture system or, more in general, µ*g* conditions as a tool for the screening of drugs targeting mechano-signaling pathways.

## Methods

### Renal progenitor cell cultures

Human RPCs were obtained and cultured as previously described [13, 37], in agreement with the Ethical Committee on human experimentation at the Azienda Ospedaliero-Universitaria Careggi in Florence, Italy (project identification code 17155/CAM_BIO). Three primary cultures from male donors, aged 54–72 years, underwent CD133 and CD24 expression analysis by FACS [37, 38] and cultured in EGM-MV medium (Lonza, Basel, Switzerland) supplemented with 20% fetal bovine serum (FBS) (HyClone, GE Healthcare Life Sciences, Logan, UT, USA). This culture medium allows the specific amplification of the CD133+/CD24+ renal cell population, as previously reported by our research group [39].

### Rotating cell culture system: cell exposure to modeled microgravity

Microgravity is a condition, especially in space orbit, where the force of gravity is so weak that weightlessness results. This condition of mechanical unloading changes the pattern of external forces acting on a cell, offering a unique opportunity for investigating mechanobiology aspect of living cells, cell aggregates and organoids. Microgravity conditions can be simulated on Earth using specific facilities. (https://spaceplace.nasa.gov/what-is-gravity/en/). In the present study, simulation of µg was achieved using a Synthecon (Houston, TX) Rotary Cell Culture System (RCCS), which featured a 10 ml high-aspect-ratio vessel (Additional file 1: Fig. S1a). The RCCS consists of a horizontally rotating, bubble-free disk-shaped culture vessel that is perfused through a semipermeable membrane. Cell sedimentation in the vessel is offset by the rotating fluid, creating a constant, gentle fall of cells through the medium. Both the vessel wall and the medium containing cells or 3D aggregates in suspension rotate at the same speed, producing a vector-averaged gravity defined “simulated or modeled µg” comparable with that of near-Earth free-fall orbit [16].

Microcarrier beads (Cytodex 1, Sigma-Aldrich, St. Louis, MO, USA—cat.C0646) are used as a substrate to culture adherent cells in RCCS. Specifically, for the experiments outlined in this paper, 4 × 10^6^ RPCs were resuspended in 16 ml of EGM-MV medium (Lonza, Basel, Switzerland) supplemented with 20% FBS (HyClone, GE Healthcare Life Sciences, Logan, UT, USA) in presence of 100 mg of pre-sterilized microcarrier beads. Cells and beads were plated in 8 wells of Ultra-Low attachment multiple 6-well plates (cat. CLS3471, Corning Incorporated, Corning New York, USA). They were cultured for 48 h to allow adhesion to the beads, followed by 16 h of starvation in Endothelial Basal Medium (Lonza). Finally, the beads covered with the cells were resuspended in EGM-MV medium or in differentiation medium (composed of DMEM-F12 + 10% FBS + 100 μM *all-trans* retinoic acid, ATRA) in presence or absence of 1 µg/mL cyclosporine A (CsA). These bead-cell complexes were transferred to the RCCS vessel (25 mg of beads/10 ml medium) placed inside a dedicated incubator set at 37 °C and 5% CO_2_. The rotation speed was set at 20 rpm, following the manufacturer’s suggestions and preliminary experiments, and the cells were exposed to the modeled-µ*g* conditions for 72 h. RPCs grown on beads cultured in the same culture medium but in Ultra-Low attachment 6-well plates placed on the floor (bottom) were used as controls of the standard gravity condition (1 × *g*).

### Cell proliferation, cell adhesion and cell spreading analysis

For cell proliferation experiments, RPCs cultured in 1 × *g* and modeled μ*g* conditions for 72 h were detached using a 0.25% trypsin–EDTA solution (Sigma-Aldrich, St. Louis, MO, USA) and then counted.

For cell adhesion and cell spreading analysis, RPCs cultured in 1 × *g* and modeled μ*g* were detached from the beads using 0.25% trypsin–EDTA solution (Sigma-Aldrich, St. Louis, MO, USA) and plated into multiwell plates in EGM-MV + 20% FBS at a density of 8 × 10^3^ cells/cm^2^. Cell analysis was conducted at 1, 3 or 24 h by using the EVOS XL Cell Imaging System (Thermo Fisher Scientific). To analyze cell adhesion through crystal violet staining, cells were plated as described above. After 3 h, cells were fixed for 15 min in 4% paraformaldehyde, stained with 0.1% (w/v) crystal violet for 20 min at room temperature, and then washed with Dulbecco’s Phosphate Buffer Saline (D-PBS). The stained cells were dissolved in 2% SDS for 30 min, and the absorbance was measured at 560 nm using the FLUOstar Optima Microplate Reader (BMG LABTECH, Ortenberg, Germany).

### Cell viability assays

For live/dead cell imaging, cells were stained with 2 µg/mL Calcein-AM (Invitrogen, Carlsbad, CA, USA) and 1 µg/mL Propidium Iodide (PI) (Miltenyi Biotec S.r.l., Bergisch Gladbach, Germany) for 20 min at 37 °C. Images were captured using the Leica SP8 STED 3X confocal microscope (Leica Microsystems, Wetzlar, Germany).

To evaluate apoptosis via flow cytometry with Annexin-V/ Propidium Iodide (PI) staining, allowing visualization of early and late stages of apoptosis, single-cell suspensions were prepared after exposure to both 1 × *g* and modeled μ*g* conditions. Cells were washed with cold D-PBS and costained with 1 µg/mL PI (Miltenyi Biotec) and Annexin V-APC (Thermo Fisher Scientific) following the manufacturer’s instructions. Samples were then acquired using the MACS Quant Analyzer and analyzed with the Flowlogic software (both from Miltenyi Biotec). Annexin V-APC was excited by a 635 nm laser line, while PI was excited by a 488 nm laser line.

### Quantitative RT-PCR

mRNA was extracted using RNeasy Microkit (Qiagen, Hilden, Germany) and retrotranscribed using TaqMan Reverse Transcription Reagents (Thermo Fisher Scientific, Waltham, MA, USA). Quantitative RT-PCR was performed as previously described [13, 38] using the following commercially available Assay on Demand kits (Thermo Fisher Scientific): Rock1 (Hs01127701_m1), Rock2 (Hs00178154_m1), nephrin (NPHS1) (Hs00190446_m1), BmI1 (Hs00995536_m1), Six2 (Hs00232731_m1).

### Immunofluorescence and confocal microscopy

Immunofluorescence and confocal microscopy were performed on RPCs cultured on Cytodex microcarrier beads using a Nikon epifluorescence microscope (Nikon, Florescence, Italy) and a Leica SP8 confocal microscope (Leica Microsystems, Wetzlar, Germany), respectively. Cells were fixed for 10 min with 4% paraformaldehyde. The following antibodies were used: anti-paxillin (ab32084, dilution 1:250, Abcam, Cambridge, UK), anti-nephrin (AF4269, dilution 1:50, R&Dsystem, Minneapolis, MN, USA), anti-tubulin (05-829, dilution 1:100, Millipore, Billerica, MA, USA), anti-vimentin (MAB1681, dilution 1:100, Millipore, Billerica, MA, USA), anti-nestin (ab5922, dilution 1:100, Millipore, Billerica, MA, USA), anti-six2 (H00010736-M01, dilution 1:100, Abnova, Taipei, Taipei, Taiwan). Staining with Alexa Fluor 546 phalloidin (Cat# A22283, dilution 1:100, Life Technologies, Monza, Italy) was performed following manufacturer’s instructions. Double immunolabelling was carried out as previously described [13]. Alexa-Fluor secondary antibodies were from Molecular Probes (Thermo Fisher Scientific) and Millipore (Billerica, MA, USA). Nuclei were counterstained with 1 μg/ml 4′,6-diamidino-2-phenylindole (DAPI, Thermo Fisher Scientific).

### Flow cytometry analysis

The expression of surface markers CD133 and CD24 was evaluated by flow cytometry analysis using the following antibodies and isotypes: CD133/2 (130-090-851 from Miltenyi Biotec), CD24 (clone SN3, SC-19585 from Santa Cruz), mouse IgG1, (130-106-545, Miltenyi Biotec) and mouse IgG2b, (130-106-547, Miltenyi Biotec), as previously described [38].

### Image analysis

The intensity of phalloidin and nephrin staining was quantified with ImageJ, by measuring raw integrated densities of the signal. Background raw integrated densities were subtracted, and this net integrated density was then normalized to the total measured cell area. Cell area was quantified with ImageJ. The number of cells analyzed is specified in the figure legends.

### Statistical analysis

The results were expressed as the mean ± SEM of the experiments reported in the figure legends. Group comparisons were performed using the Mann–Whitney test, as appropriate. Statistical analysis was performed using OriginPro statistical software (OriginLab Corporation, MA, USA). A *p* value < 0.05 was considered statistically significant.

## Results

### Cytodex microbeads support expansion of renal progenitors as undifferentiated cells

**Fig. 1:**
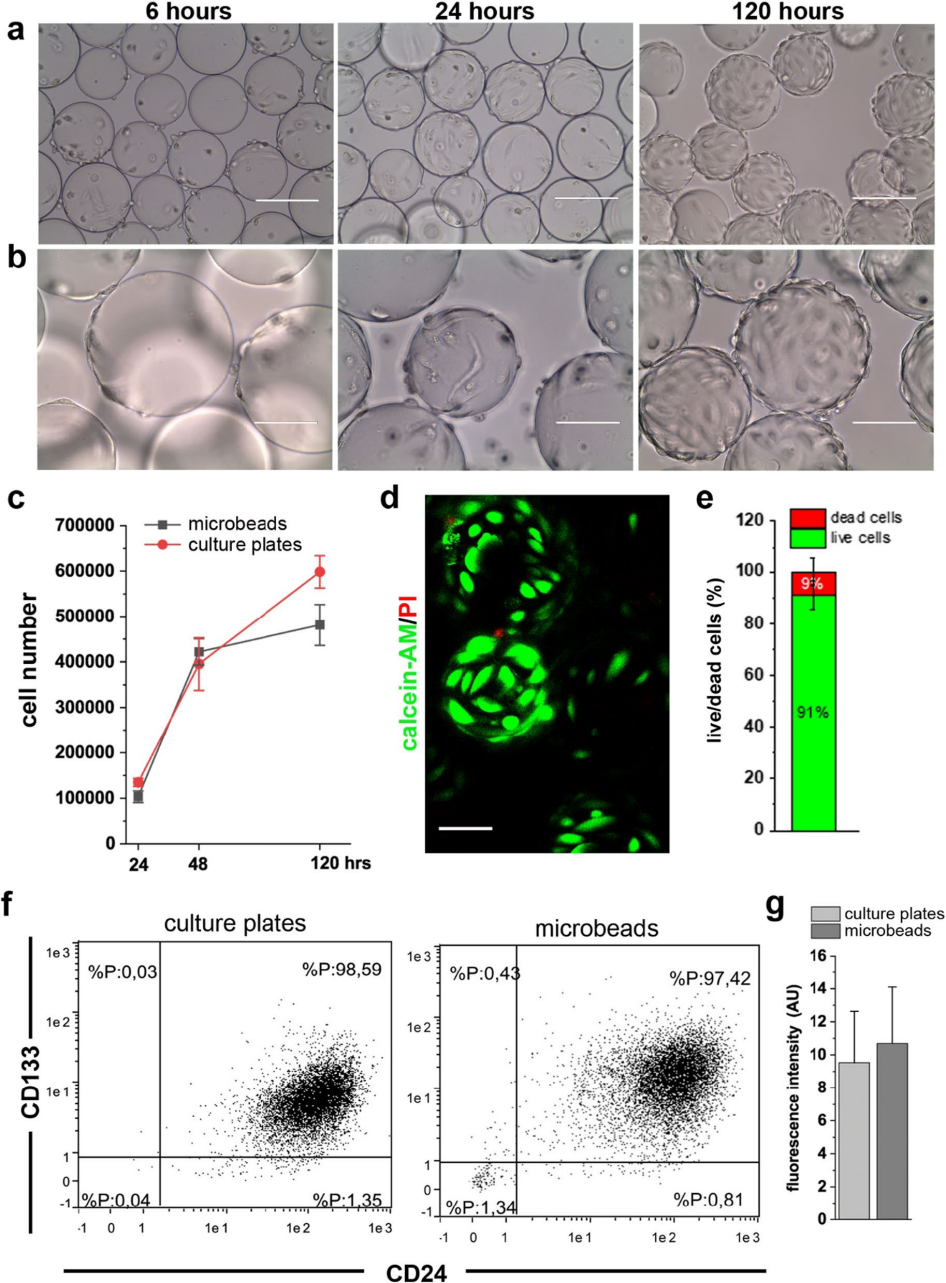
Culture on Cytodex microbeads supported renal progenitor cells expansion and maintenance of their phenotype. **a**, **b** Phase-contrast images of RPCs cultured on Cytodex microbeads at different time points. BAR = 200 μm for (**a**) and BAR = 100 μm for (**b**). **c** Growth curve of RPCs cultured on culture plates and on Cytodex microbeads. Data are expressed as mean ± SEM of three independent experiments. **d** Representative confocal image of Calcein-AM (green fluorescence) and PI (red) staining in RPCs after 120 h of culture on Cytodex microbeads. BAR = 75 μm. **e** Percentage of dead and live RPCs quantified from confocal images of Calcein-AM/PI staining. Data are expressed as mean ± SEM of three independent experiments. **f** Representative flow cytometry analysis of CD133 and CD24 staining of RPCs before (left) and after (right) 120 h of culture on Cytodex microbeads. **g** Mean fluorescence intensity of CD133 flow cytometry staining. Data are expressed as the mean ± SEM of three independent experiments

To culture adherent cells in RCCS, the facility used to model µ*g*, Cytodex microcarrier beads are required as a substrate for cell adhesion. To ensure that modification in the composition and topography of the substrate alone did not induce any changes in the cells, we carried out preliminary experiments with RPCs cultured on the beads. RPCs demonstrated the capacity to spontaneously adhere to the carrier beads after 3–6 h in culture (Fig. 1a, b). It was evident that on the convex spherical structures of the beads, the cell body was pulled upward, minimizing the contact area with the substrate (Fig. 1a, b). Over the next 120 h, RPCs on the Cytodex microbeads proliferated (Fig. 1c), covering each bead with a single layer of viable cells (Fig. 1d, e). Moreover, culture of RPCs on the beads did not induce modifications in the expression of CD133 and CD24, the two stem cell markers characteristic of this subpopulation of cells (Fig. 1f, g), nor did it affect the expression of other stem cell markers (Additional file 1: Fig. S1b). These data demonstrate that the substrate composition and curvature support growth and viability of RPCs while maintaining their progenitor characteristics.

### Modeled microgravity conditions alter cytoskeleton organization and formation of focal adhesions in renal progenitor cells

**Fig. 2:**
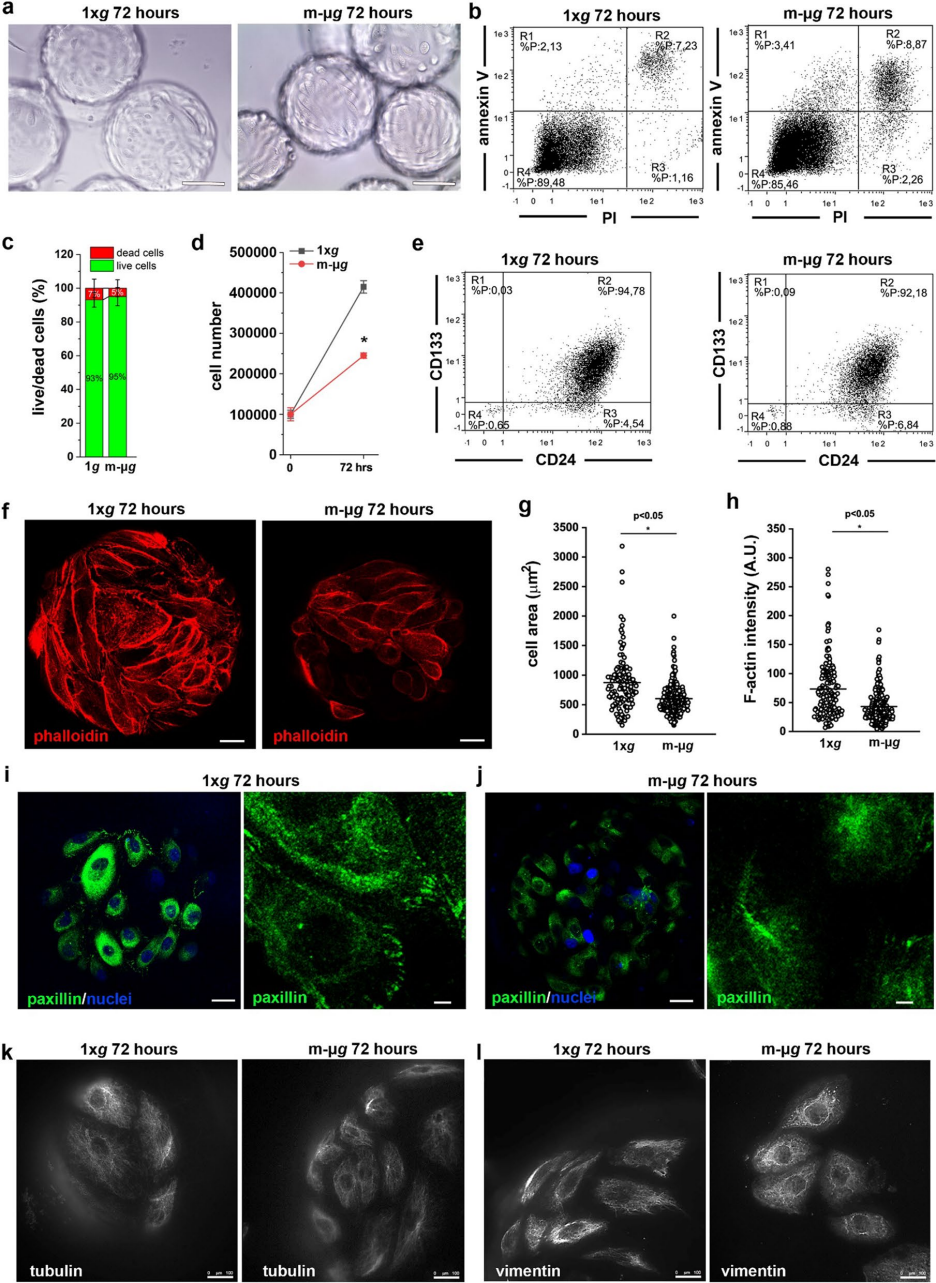
Modeled μ*g* conditions reduced proliferation and altered the cytoskeleton of renal progenitor cells. **a** Phase-contrast images of RPCs grown on Cytodex microbeads at 1 × *g* (top) and in modeled μ*g* (bottom) conditions. BAR = 100 μm. **b** Representative flow cytometry analysis of Annexin V/PI staining of RPCs cultured for 72 h at 1 × *g* (left) and in modeled μ*g* (right) conditions. **c** Percentage of dead and alive RPCs quantified from confocal images of Calcein-AM/PI staining. Data are expressed as mean ± SEM of two independent experiments. **d** Growth curves obtained culturing RPCs for 72 h at 1 × *g* and in modeled μ*g* conditions. Data are expressed as mean ± SEM of three independent experiments. **e** Representative flow cytometry analysis of CD133 and CD24 staining of RPCs cultured for 72 h at 1 × *g* (left) and in modeled μ*g* (right) conditions. **f** Confocal images of phalloidin (red fluorescence) staining in RPCs cultured for 72 h at 1 × *g* (left) and in modeled μ*g* (right) conditions. BAR = 25 μm. **g** Measurement of RPC cell area from images as showed in panel (**f**). At least 50 cells for each condition were analyzed from three independent experiments. **h** Quantification of phalloidin staining of RPCs cultured for 72 h at 1 × *g* and in modeled μ*g* conditions. At least 50 cells for each condition were analyzed from three independent experiments. **i**, **j** Confocal images of paxillin (green) and nuclei (DAPI, blu) staining in RPCs cultured for 72 h at 1 × *g* (**i**) and in modeled μ*g* (**j**) conditions. BAR = 25 μm and 5 μm, respectively. **k** Images of tubulin (white fluorescence) staining in RPCs cultured for 72 h at 1 × *g* (left) and in modeled μ*g* (right) conditions. BAR = 100 μm. **l** Images of vimentin (white fluorescence) staining in RPCs cultured for 72 h at 1 × *g* (left) and in modeled μ*g* (right) conditions. BAR = 100 μm. Statistical analysis was performed using the Mann–Whitney test. m-μ*g*: modeled microgravity

RPCs spontaneously adhering to the carrier beads where then introduced into the RCCS and cultured for 72 h in modeled μ*g* condition*.* In this condition, RPCs remained anchored to the microcarrier beads (Fig. 2a) without any differences in the percentage of dead cells and cells in early and late phases of apoptosis compared to cells cultured on beads at 1 × *g*, as assessed by Annexin V/PI staining (Fig. 2b) (13.4 ± 3% vs 14.3 ± 4%, 1 × *g* vs modeled μ*g,* NS). Quantification of immunofluorescence staining for Calcein-AM/PI confirmed these results (Fig. 2c). However, the number of cells obtained after 72 h of culture in modeled μ*g* condition was significantly lower in comparison with the 1 × *g* condition (Fig. 2d), suggesting that unloading reduced the proliferation ability of RPCs. On the contrary, modeled μ*g* condition did not induce modification in the expression of the CD133 and CD24 stem cell markers (96.7 ± 2.7% vs 93.9 ± 2.4%, 1 × *g* vs modeled μ*g*, NS) (Fig. 2e).

In several cell types, the most apparent cellular changes that occur following exposure to a μ*g* environment are alterations of cell shape and adhesion/migration, reflecting modifications in cytoskeletal structures [20, 23, 27, 29, 40, 41]. Accordingly, RPCs maintained in modeled μ*g* conditions showed significant changes both in cell shape and in the organization of the actin cytoskeleton, as demonstrated by staining with fluorescent phalloidin (Fig. 2f). Indeed, in comparison with cells grown on Cytodex beads at 1 × *g*, the cells exposed to modeled μ*g* conditions appeared smaller (Fig. 2g) with a decreased density of F-actin fibers (Fig. 2h), whose network was disorganized (Fig. 2f). Moreover, staining with anti-paxillin antibody demonstrated that unloading conditions severely reduced the number of cells showing focal adhesions, appearing as paxillin spots located at the cell edges only in the 1 × *g* conditions (Fig. 2i, j).

These results prompted us to analyze the effect of modeled μ*g* on the other major components of the cytoskeleton, namely microtubules and intermediate filaments. Staining with anti-tubulin antibody revealed a rarefaction of the microtubule network at the cell periphery in the cells exposed to modeled μ*g*, compared to 1 × *g* controls (Fig. 2k). Indeed, in 2D images, the cells exposed to unloading conditions appeared smaller. It can be hypothesized that, following the reorganization of the microtubule network, the cell ability to spread on the substrate decreased.

The exposure to modeled μ*g* conditions also induced a marked rearrangement of the intermediate filaments. As shown by anti-vimentin antibody staining in cells exposed to modeled μ*g*, compared to the 1 × *g* controls, intermediate filaments appeared fragmented and condensed around the nucleus (Fig. 2l).

### Modeled microgravity transiently hampered renal progenitor cell adhesion and spreading capacity

**Fig. 3:**
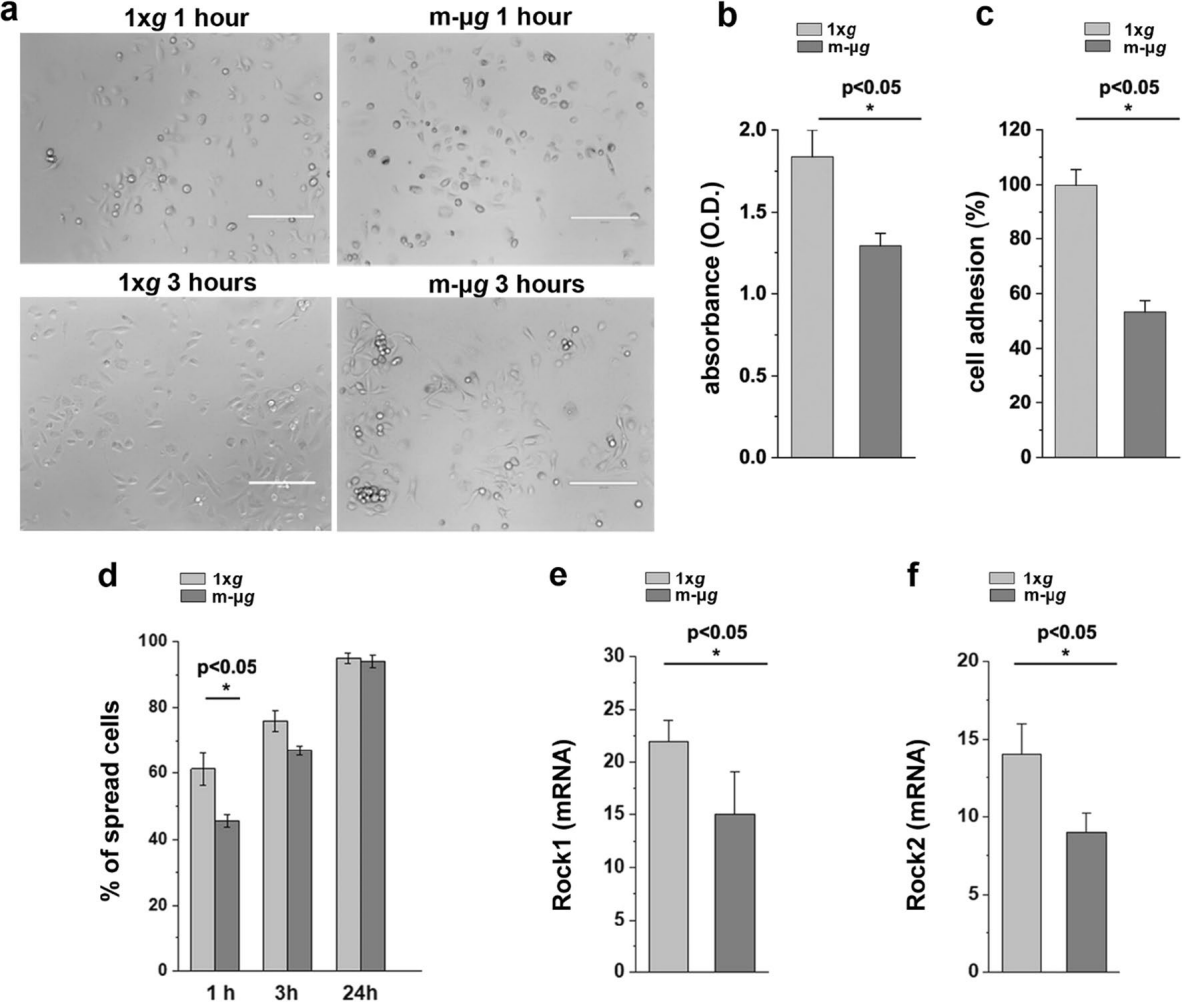
Modeled microgravity transiently hampered renal progenitor cell adhesion and spreading capacity. **a** Phase-contrast images of RPCs taken 1 and 3 h after 72 h of exposure to 1 × *g* (left) or modeled μ*g* (right). BAR = 400 μm. **b** Adherent RPCs quantified 3 h after 72 h exposure to 1 × *g* and modeled μ*g* assessed by Crystal violet staining. Data are expressed as mean ± SEM of three independent experiments. **c** Percentage of RPCs adhering to the culture plates 3 h after 72 h exposure to 1 × *g* and modeled μ*g* over total cells present in the microscopy field*.* At least 10 representative images from each condition from three independent experiments were analyzed. **d** Percentage of RPCs displaying a spread morphology on the culture plates at 1, 3 and 24 h after 72 h exposure to 1 × *g* or modeled μ*g*. At least 10 representative images from each condition were analyzed in three independent experiments. Data are expressed as mean ± SEM. **e** Rock1 and Rock 2 **f** mRNA expression in RPCs exposed to 1 × *g* or modeled μ*g*. Data are expressed as mean ± SEM of two independent experiments. Statistical analysis was performed using the Mann–Whitney test. m-μ*g*: modeled microgravity

Disorganization of the cytoskeleton and lack of paxillin may decrease cell adhesion by inhibiting focal adhesion assembly. Therefore, we analyzed the adhesion and spreading capacity of renal progenitor cells following 72 h of exposure to unloading conditions. To this aim, RPCs were exposed to modeled μ*g* or maintained at 1 × *g* for 72 h. Subsequently, the cells were detached from the microcarrier beads, plated in standard culture plates and evaluated during the recovery period. Exposure to modeled μ*g* induced a decreased adhesion rate compared to RPCs maintained at 1 × *g*, as assessed by both crystal violet adhesion assay and adherent cell count 1 and 3 h after plating (Fig. 3a–c). Moreover, during the initial 3 h following the return to normal gravity, RPCs exhibited a decrease in the ability to spread on the substrate (Fig. 3d) that was almost completely recovered after 24 h (Fig. 3d). These functional alterations were concurrent with a reduction in the expression of Rock1 and Rock2 (Fig. 3e, f). In conclusion, exposure to modeled μ*g* induces changes in cell adhesion that are largely reversible upon return to normal gravity.

### Mechanical forces are necessary for the differentiation of renal progenitor cells into podocytes

**Fig. 4:**
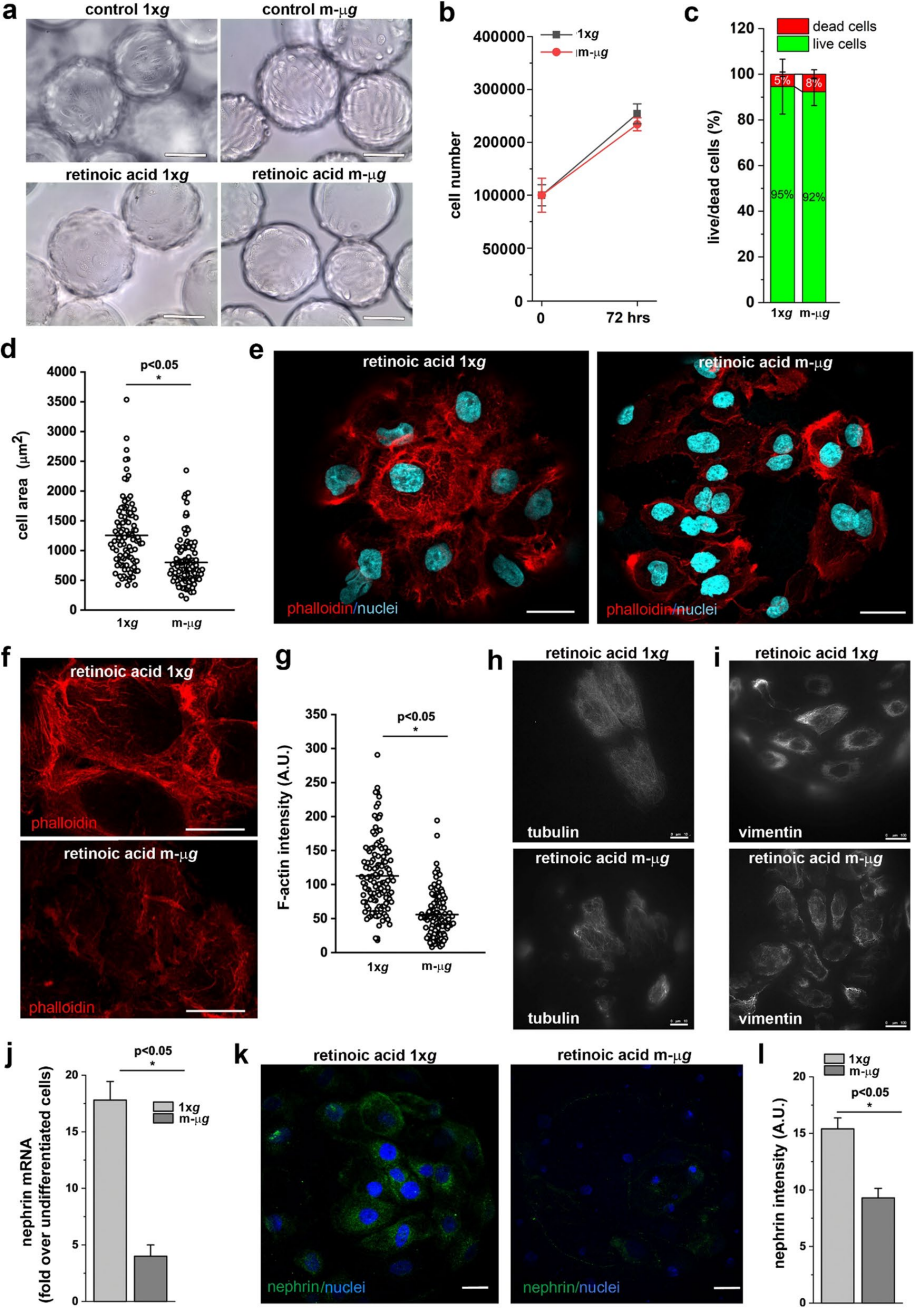
Modeled microgravity impaired renal progenitor cell differentiation into podocyte. **a** Building upon this knowledge and our findings regarding the effects of modeled μ*g* conditions on RPC morphology and function, we sought to investigate the influence of mechanical forces on the differentiation of RPCs into podocytes in the context of reduced mechanical stress due to the unloading conditions modeled by RCCS. modeled μ*g* conditions. BAR = 100 μm. **b** Growth curves of RPCs cultured in presence of retinoic acid for 72 h at 1 × *g* and in modeled μ*g* conditions. Data are expressed as mean ± SEM of three independent experiments. **c** Quantification of the percentage of dead and alive cells determined from confocal images of Calcein-AM/PI staining. Data are expressed as mean ± SEM of three independent experiments. **d** Measurement of RPC cell area from images. At least 40 cells for each condition were analyzed from three independent experiments. **e** Confocal images of phalloidin (red fluorescence), and DAPI (light blue) staining in RPCs cultured for 72 h in presence of retinoic acid at 1 × *g* (right) and in modeled μ*g* (left) conditions. BAR = 25 μm. **f** Higher magnification of phalloidin staining in RPCs cultured for 72 h in the different conditions. BAR = 25 μm. **g** Quantification of phalloidin staining. At least 40 cells for each condition were analyzed from three independent experiments. **h** Images of tubulin (white fluorescence) staining in RPCs cultured for 72 h in the different conditions. BAR = 10 μm. **i** Images of vimentin (white fluorescence) staining in RPCs cultured for 72 h in the different conditions. BAR = 100 μm. **j** Expression of nephrin mRNA in RPCs cultured for 72 h in presence of retinoic acid at 1 × *g* and in modeled μ*g* conditions. Data are expressed as mean ± SEM of three independent experiments. **k** Confocal images of nephrin expression (green fluorescence) in RPCs cultured for 72 h in presence of retinoic acid at 1 × *g* and in modeled μ*g* conditions. BAR = 25 μm. **l** Quantification of nephrin staining. Data are expressed as mean ± SEM of three independent experiments. Statistical analysis was performed using the Mann–Whitney test. m-μ*g*: modeled microgravity

In many contexts, mechanical forces external to the cell have been shown to impact stem cell differentiation. These forces include shear stress from fluid flow and more localized mechanical cues such as cell density, shape and elasticity of the surrounding extracellular matrix [42]. Building upon this knowledge and our findings regarding the effects of modeled μ*g* conditions on RPC morphology and function, we sought to investigate the influence of mechanical forces on the differentiation of RPCs into podocytes in the context of reduced mechanical stress due to the unloading conditions modeled by RCCS. To this aim, we exposed RPCs to modeled μ*g* for 72 h in a culture medium containing 100 μM *all-trans* retinoic acid (ATRA), a known inducer of podocyte differentiation [43]. Cells treated with ATRA and maintained in 1 × *g* condition were used as control. In the presence of ATRA, RPCs exhibited a flattened morphology in both 1 × *g* and modeled μ*g* conditions (Fig. 4a). Differentiation with ATRA led to a strong reduction in cell proliferation compared to undifferentiated cells, with no discernible difference induced by modeled μ*g* conditions (Fig. 4b). Similarly, there was no difference in the percentage of apoptotic cells between 1 × *g* and modeled μ*g* condition (Fig. 4c). Podocyte function relies on the maintenance of a correct cytoskeletal architecture and changes in podocyte actin dynamics directly affect kidney function. Therefore, we examined the effect of weightlessness conditions on the organization of F-actin, by staining with phalloidin, as well as tubulin and vimentin by staining with their respective anti-protein antibodies. The cells differentiated in 1 × *g* condition increased in size up to 1,254 ± 583 μm^2^ (mean ± SD, Fig. 4d) compared to undifferentiated cells (mean area 889 ± 454 μm^2^, Fig. 2g) and developed actin-rich finger-like structures around their periphery (Fig. 4e, left and 4f, top). On the contrary, cells obtained in modeled μ*g* conditions displayed a smaller cell size (mean area 798 ± 400 μm^2^) (Fig. 4d) and an altered cytoskeleton architecture with the absence of finger-like structures (Fig. 4e, right and 4f, bottom), and reduced density of F-actin, as revealed by staining (Fig. 4g). Moreover, these cells exhibited alterations in the microtubule and intermediate filament networks which, together with the alterations observed in the actin microfilaments, highlight a marked remodeling of the major cytoskeletal components, resulting in evident morphological alterations (Fig. 4h, i). More importantly, while differentiation of RPCs at 1 × *g* conditions resulted in the upregulation of the expression of the podocyte marker nephrin, the induction of nephrin expression was impaired in modeled μ*g* conditions (Fig. 4j–l). Similar results were obtained for nestin (Additional file 1: Fig. S1d, e). These findings suggest that exposure to mechanical forces is necessary for the generation of fully differentiated podocytes with a specialized cytoskeletal organization.

### Stabilization of cytoskeleton with drugs rescues the differentiation of renal progenitor cells into podocytes in modeled microgravity conditions

**Fig. 5:**
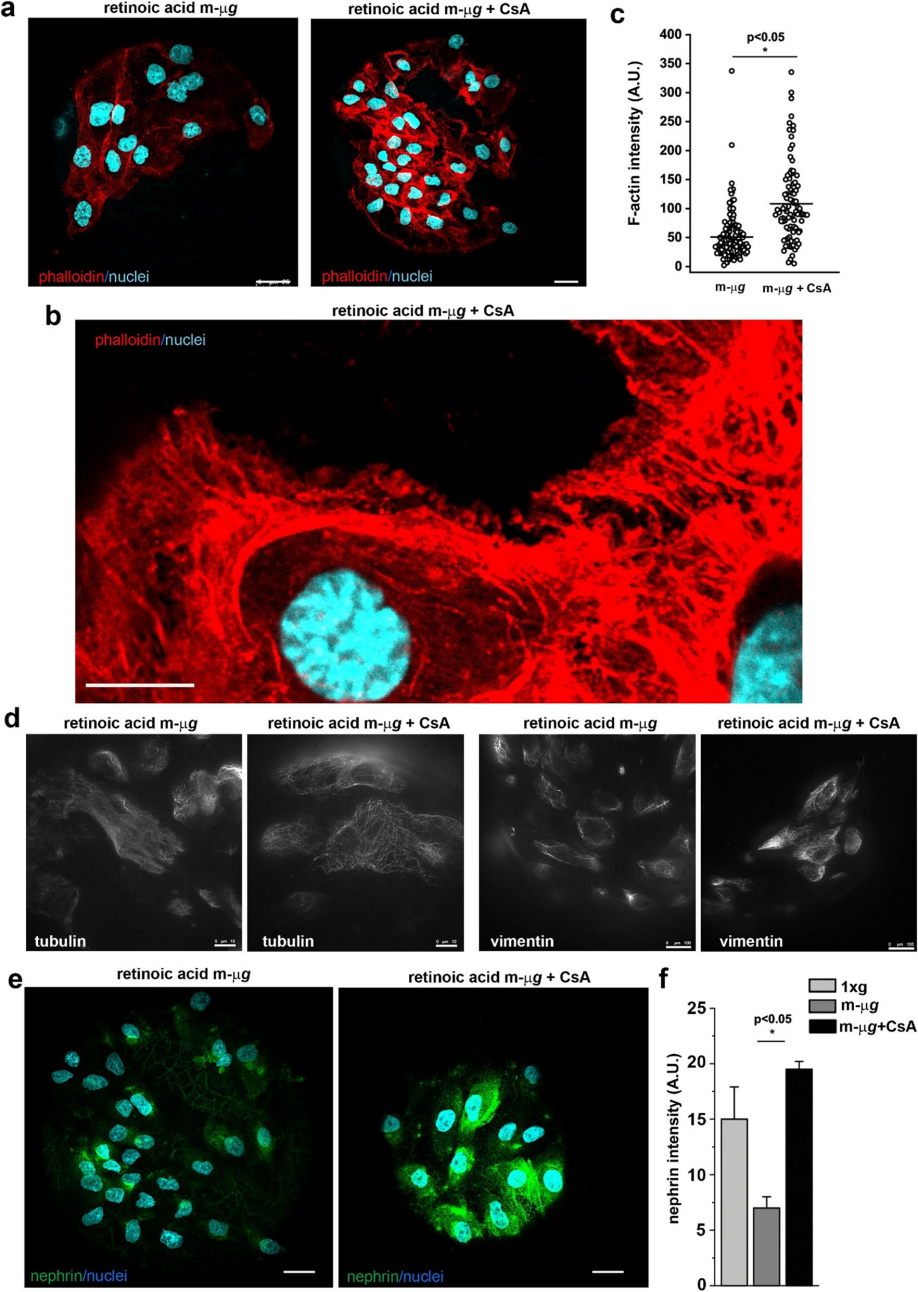
The presence of Cyclosporine A during modeled microgravity exposure restored renal progenitor cell differentiation into podocyte. **a** Confocal images of phalloidin (red fluorescence), and DAPI (light blue) staining in RPCs cultured for 72 h with retinoic acid in modeled μ*g* conditions in absence (left) or in presence (right) of CsA. BAR = 25 μm. **b** Higher magnification view of phalloidin (red fluorescence), and DAPI (light blue) staining in RPCs cultured for 72 h with retinoic acid in modeled μ*g* conditions in presence of CsA. BAR = 10 μm. **c** Quantitative analysis of phalloidin staining in RPCs cultured for 72 h with retinoic acid in modeled μ*g* conditions in absence or in presence of CsA. At least 50 cells for each condition were analyzed from two independent experiments. **d** Images of tubulin and vimentin (white fluorescence) staining in RPCs cultured for 72 h with retinoic acid in absence or presence of CsA in modeled μ*g* conditions. BAR = 10 μm for tubulin and BAR = 100 μm for vimentin. **e** Confocal images of nephrin expression (green fluorescence) and DAPI (light blue) staining in RPCs cultured for 72 h with retinoic acid in modeled μ*g* conditions in absence (left) or in presence (right) of CsA. BAR = 25 μm. **f** Quantification of nephrin staining in RPCs cultured for 72 h with retinoic acid in modeled μ*g* conditions in absence or in presence of CsA. Data are expressed as the mean ± SEM of two independent experiments. Statistical analysis was performed using the Mann–Whitney test. m-μ*g*: modeled microgravity

Recent reports have indicated that compounds enhancing actin polymerization in injured podocytes can improve renal health both in transient kidney disease and in chronic kidney disease models [44]. Additionally, the immunosuppressive agent cyclosporine A (CsA) exerts its anti-proteinuric effect through the direct stabilization of the actin cytoskeleton in podocytes [7]. Consequently, we were interested in investigating whether treating RPCs with CsA during the differentiation in the RCCS could rescue the observed phenotype. To test this hypothesis, we differentiated RPCs with ATRA in modeled μ*g* conditions for 72 h in presence or absence of CsA. The presence of CsA during the exposure of RPCs to modeled μ*g* conditions was sufficient to partially restore the altered organization of the actin, tubulin and vimentin cytoskeleton induced by mechanical unloading (Fig. 5a–d). Even more interestingly, CsA treatment increased the expression of nephrin (Fig. 5e, f) and nestin (Additional file 1: Fig. S1e). These results validate the notion that alterations of the correct assembly of the cytoskeleton, which hamper RPC differentiation, can be a direct target for actin stabilizing drugs. Furthermore, we propose the RCCS system as an in vitro tool for evaluating the efficacy of cytoskeleton-targeted drugs, offering an alternative to animal testing.

## Discussion

Alterations in mechanical forces within the glomerulus are effectors of podocyte injury, which is the unifying trigger of podocytopathies [4, 5]. The first sign of podocyte damage is the rearrangement of the F-actin cytoskeleton, which correlates with the near-complete loss of their ability to apply force to the substrate [45]. Subsequently, glomerular damage may progress due to both podocyte loss and a defective regenerative response mediated by renal progenitors [4, 5]. Recently, mechanical stimuli have emerged as regulators of renal progenitor behavior through modification of F-actin organization [13], although their impact on this cell population is likely still underestimated. In this study, we used the RCCS to induce modification in the mechanical environment by modeling μg condition. We demonstrated that this condition, also referred to as mechanical unloading, induces cytoskeleton alteration in RPCs and that a correct assembly of microfilaments, microtubules and intermediate filaments is necessary to support renal progenitor differentiation into podocytes. Moreover, we provide evidence that RCCS and other systems capable of modeling mechanical cues in the cell microenvironment could represent a valuable in vitro tool for the screening of “mechano” drugs of interest to nephrologist.

Cytoskeleton alterations appeared as the first response of RPCs to unloading conditions produced by RCCS, consistent with observations in other cell types [20, 23, 27, 46–49]. To simulate μg within the RCCS, RPCs were cultured on the surface of cross-linked dextran microbeads. These beads, differing from the standard conditions used for renal progenitor culture, vary in both the chemical composition (dextran vs polystyrene) and the geometry (convex vs planar surface) of the substrate, two material-dependent physical cues known to potentially affect the physiology of various cell types [50, 51]. However, our results clearly demonstrate that the modifications observed in renal progenitor phenotype were induced solely by modeled μ*g* conditions, with no discernible effects attributed to the nanotopographical features or the composition of the beads alone.

In parallel with cytoskeleton alterations, we observed a reduction in the assembly of focal adhesions, cellular components notoriously involved in converting mechanical cues into intracellular signals, thereby regulating downstream cellular activities [52]. In particular, modification of the mechanical microenvironment resulted in a transient reduction in RPC adhesion and spreading capacity, a phenomenon described in other cell types [53]. Notably, recent studies have demonstrated that vimentin, which contributes to cells resilience against mechanical stress [54], generates with actin and microtubules the forces required for cell adhesion. More specifically, vimentin regulates stress generation by adherent cells through an interplay with actin stress fibers [55]. We can speculate that alteration of the mechanical forces within the glomerulus in vivo may lead to cytoskeleton alterations and the detachment of renal progenitors from the basement membrane. Intriguingly, detached yet viable renal progenitors shed into the urine have been reported in patients with kidney disorders [56, 57], kidney transplant recipients [58] and healthy subjects [59, 60]. This suggests that changes in cytoskeletal organization and impaired progenitor adhesion to the basement membrane of the Bowman capsule or to the glomerular basement membrane (GBM), resulting from alterations in mechanical forces, may be a pivotal step in the development and progression of glomerulosclerosis.

Furthermore, our study demonstrates that the cytoskeletal alterations induced by altered mechanical conditions were associated with hampered renal progenitor differentiation into podocytes, as evidenced by the reduced expression of the podocyte markers nephrin and nestin. Overall, our findings strengthen the concept that changes in mechanical cues can initiate a pathophysiological process in the glomerulus [3, 4] not only altering podocyte actin cytoskeleton, but also extending the detrimental effect to the renal progenitor population. Various types of stem cells have shown altered differentiation capacity in both modeled and microgravity conditions [20, 34, 61, 62]. It is well established that microgravity-induced alterations in cell differentiation processes can affect important biological processes, including wound healing, tissue regeneration [29–31], the homeostasis, turnover and function of various tissues and systems such as bone, muscle, cartilage, the immune system, and more [63–67]. These multiple and concomitant alterations are part of the mechanisms of adaptation to spaceflight observed in astronauts [68, 69]. There is limited data available regarding the renal response to space conditions, with no information on the effects on the kidney progenitor population. To the best of our knowledge, this study represents the first attempt to shed light on the effects induced by microgravity in RPCs, which is critical for assessing health risks during long-duration space missions. However, a more comprehensive study, potentially using single-cell RNA sequencing techniques together with combining various cell types into organoids or spheroids, would be necessary to gain mechanistic insights into the signalling pathways involved in RPC responses to modeled μ*g* conditions.

Our research on the effects of unloading conditions on the behavior of RPCs appears of interest in other contexts as well. Firstly, understanding the mechanical factors that contribute to maintaining the undifferentiated phenotype of renal progenitors or specifying their fate decisions can help in engineering defined, synthetic niche systems with complete control of mechanical, matrix and soluble cues for the long-term culture of functional kidney stem cells or organoids both in space and on Earth [15]. Secondly, elucidating the role of the cytoskeleton in maintaining renal progenitor regenerative capacity strengthens the concept that actin, and probably other cytoskeleton components, could represent druggable targets for kidney diseases, as recently reported [70]. Finally, employing protocols for cell differentiation in the RCCS could represent an in vitro tool to evaluate the efficacy of cytoskeleton-targeted drugs, offering an alternative to animal testing.

## Conclusions

By using modeled μg conditions, we have demonstrated that the regenerative capacity of RPCs is regulated by mechanical stimuli that impact the organization of the actin cytoskeleton. Dysregulated actin cytoskeleton in different renal cell types is a common manifestation of several kidney diseases. Consequently, research in microgravity conditions could be instrumental in devising targeted treatments that engage with the actin network to treat kidney diseases. Additionally, it could serve as a platform for testing potential countermeasures to mitigate the harmful effects of prolonged exposure to a lack of gravitational force during extended spaceflights.

### Supplementary Information

**Additional file 1: Figure S1.** **a** The RCCS equipment used in the study. This cell culture system is a modification of the original Rotating Wall Vessel developed by NASA to simulate microgravity conditions and it is commercially available through Synthecon Cellon S.r.l. (Strassen Luxemburg). RCCS consists of a relatively large container (vessel) with gas exchange membrane. The culture chamber with diffusion gas exchange is completely filled with culture medium. The fluid mass rotation is accomplished by the vessel rotating horizontally around its axis, randomizing the gravitational forces acting on the cell surface. Indeed, as the vessel rotates, cells are subjected by a constantly changing angular gravity vector. **b** Expression of stem cell markers assessed in RPCs cultured for 72 h at 1 × g (green) and in modeled μg (pink) conditions by RT-PCR. Data are expressed as the mean ± SEM of two independent experiments. **c** Representative image of Six2 staining in RPCs. BAR = 25 μm. **d** Images of nestin staining in RPCs cultured on culture plates in presence or absence of retinoic acid. **e** Images of nestin staining in RPCs cultured in the different conditions. BAR = 25 μm.

## Data Availability

The datasets used and/or analyzed during the current study are available from the corresponding author on reasonable request.

## Abbreviations

RPC: Renal progenitor cells
RWV: Rotating wall vessel
RCCS: Rotating cell culture system
RPM: Random positioning machine
FBS: Fetal bovine serum
D-PBS: Dulbecco modified phosphate buffer saline
ATRA: *All-trans* Retinoic acid
1 × *g*: Standard gravity
m-μ*g*: Modeled microgravity
PI: Propidium iodide
SEM: Standard error of mean
CsA: Cyclosporine A
