# Supplementary material for: Modeled microgravity unravels the roles of mechanical forces in renal progenitor cell physiology
Source: Stem Cell Res Ther. 2024 Jan 17;15:20. doi: 10.1186/s13287-024-03633-3 (PMC10795253; doi:10.1186/s13287-024-03633-3)
Supplement: Supplementary file 1 — Additional file 1: Figure S1. a The RCCS equipment used in the study. This cell culture system is a modification of the original Rotating Wall Vessel developed by NASA to simulate microgravity conditions and it is commercially available through Synthecon Cellon S.r.l. (Strassen Luxemburg). RCCS consists of a relatively large container (vessel) with gas exchange membrane. The culture chamber with diffusion gas exchange is completely filled with culture medium. The fluid mass rotation is accomplished by the vessel rotating horizontally around its axis, randomizing the gravitational forces acting on the cell surface. Indeed, as the vessel rotates, cells are subjected by a constantly changing angular gravity vector. b Expression of stem cell markers assessed in RPCs cultured for 72 h at 1 × g (green) and in modeled μg (pink) conditions by RT-PCR. Data are expressed as the mean ± SEM of two independent experiments. c Representative image of Six2 staining in RPCs. BAR = 25 μm. d Images of nestin staining in RPCs cultured on culture plates in presence or absence of retinoic acid. e Images of nestin staining in RPCs cultured in the different conditions. BAR = 25 μm. [file 13287_2024_3633_MOESM1_ESM.docx]

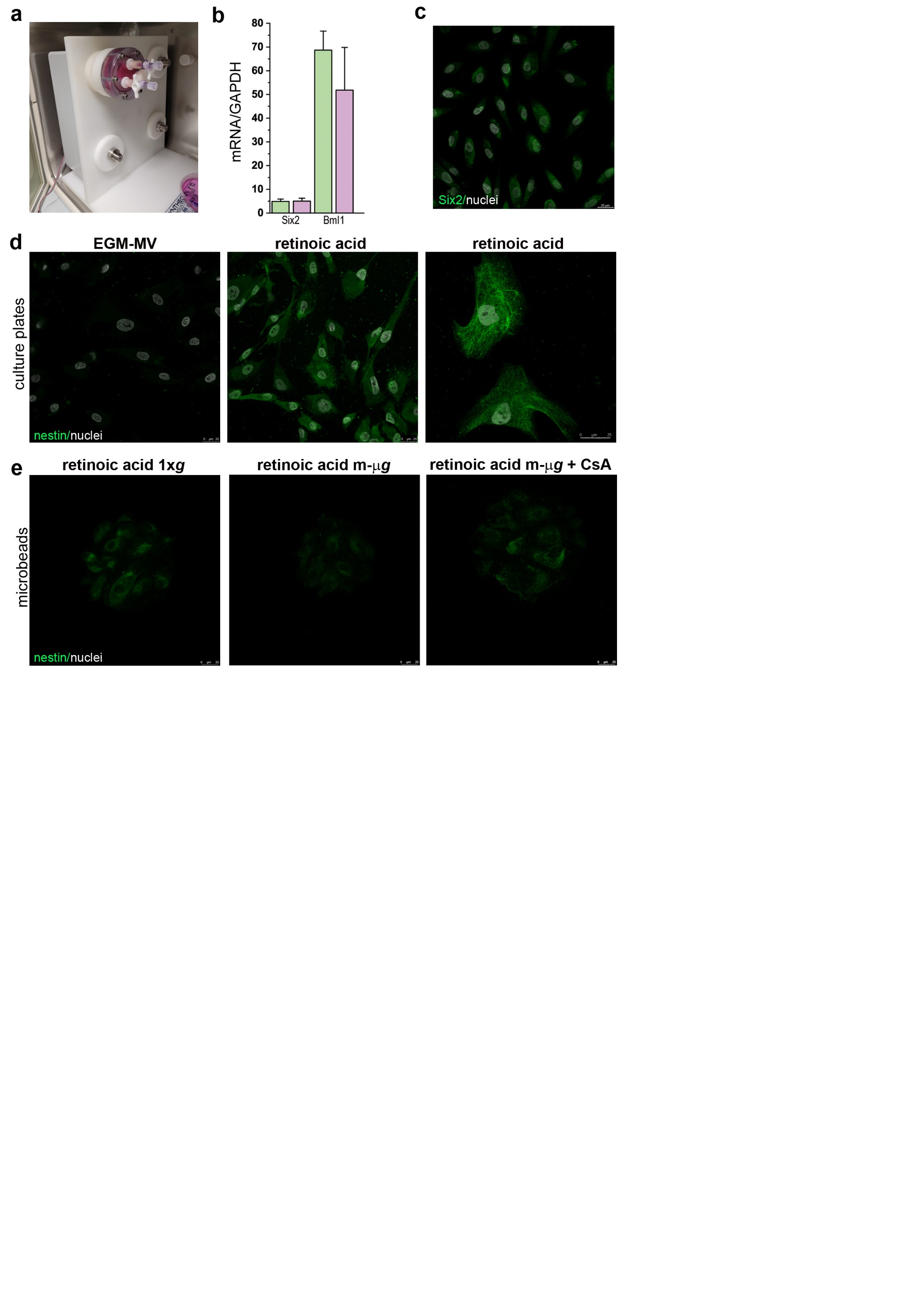


Supplementary Figure 1 (a) The RCCS equipment used in the study. This cell culture system is a modification of the original Rotating Wall Vessel developed by NASA to simulate microgravity conditions and it is commercially available through Synthecon Cellon S.r.l. (Strassen Luxemburg). RCCS consist of a relatively large container (vessel) with gas exchange membrane. The culture chamber with diffusion gas exchange is completely filled with culture medium. The fluid mass rotation is accomplished by the vessel rotating horizontally around its axis, randomizing the gravitational forces acting on the cell surface. Indeed, as the vessel rotates, cells are subjected by a constantly changing angular gravity vector. (b) Expression of stem cell markers assessed in RPCs cultured for 72 hours at 1xg (green) and in modeled μg (pink) conditions by RT-PCR. Data are expressed as the mean ± SEM of two experiments. (c) Representative image of Six2 staining in RPCs. BAR=25 μm. (d) Images of nestin staining in RPCs cultured on culture plates in presence or absence of retinoic acid. (e) Images of nestin staining in RPCs cultured in the different conditions. BAR=25 μm.
